# Dynamic Balance and Chest Mobility of Older Adults after Speleotherapy Combined with Pulmonary Rehabilitation, Endurance and Strength Training—A Prospective Study in Chronic Respiratory Diseases

**DOI:** 10.3390/ijerph191811760

**Published:** 2022-09-18

**Authors:** Sylwia Mętel, Magdalena Kostrzon, Justyna Adamiak

**Affiliations:** 1 Institute of Applied Sciences, University of Physical Education in Krakow, 31-571 Krakow, Poland; 2 ‘Wieliczka’ Salt Mine Health Resort in Wieliczka, 32-020 Wieliczka, Poland

**Keywords:** the elderly, FSST, chest expansion test, chronic respiratory diseases, subterranean therapy

## Abstract

Backgrounds: As people age, they are more likely to experience balance disturbances. Pulmonary rehabilitation is recognized as a core component in the management of older adults with chronic respiratory diseases. The aim of the study was to assess the dynamic balance and chest mobility of older adults participating in speleotherapy combined with pulmonary rehabilitation, endurance and strength training. Methods: The study group consisted of 51 older adults with chronic respiratory disorders who participated in a 3-week pulmonary rehabilitation programme in underground salt chambers in the ‘Wieliczka’ Salt Mine Health Resort. These individuals underwent the Four Square Step Test (FSST) and circumferential chest mobility measurement before and after the outpatient rehabilitation programme conducted 135 m underground. Results: Before rehabilitation in the underground salt chambers, half of the results (50%, 22 patients) were below the norm in the assessment of chest mobility between maximal inhale and exhale. The average time needed to perform FSST decreased significantly (*p* ≤ 0.05) from 10.2 ± 1.9 s before the stay to 9.1 ± 1.7 s after the stay and the average increase in chest mobility increased significantly (*p* ≤ 0.05) from 4.5 ± 5.5 cm to 5.4 ± 2.8 cm. Conclusions: Speleotherapy combined with pulmonary rehabilitation, endurance and strength training increased the dynamic balance and chest mobility of older adults with chronic respiratory diseases, as measured by the FSST and circumferential chest expansion assessment.

## 1. Introduction

The aging process of the human body weakens the ability to maintain posture control, which leads to disturbances in the balance of the body related to the vestibular, visual and proprioceptive systems and the perturbation response [1]. Approximately 28–35% of people aged 65 and over fall each year [2]. Age-related reductions in physiological capacity are most pronounced in respiratory mechanics. Patients with chronic obstructive pulmonary diseases (COPD’s) have balance impairments compared to healthy subjects, which may be related to reduced muscle strength, physical activity and exercise capacity [3,4,5] and are four-times more likely to fall than healthy peers, leading to increased morbidity and mortality [6]. What is more, they also demonstrate scapular control impairments, such as scapular elevation, internal rotation and protraction, which have negative influences on geometrical arrangements of the thorax and scapulae [7].

Over the course of the adult life span, there is a progressive increase in the rigidity of the chest wall and a decrease in the elastic recoil of the lungs. Restriction of chest expansion is often observed in pulmonary patients and is an important element in their functional status [3,8,9]. Regular physical activity effectively helps older adults improve or delay the loss of physical function and mobility while reducing the risk of fall-related injuries [10]. Pulmonary rehabilitation is recognized as a core component in the management of older adults with chronic respiratory diseases and enables healthy aging, which is a benefit for both individuals and society. Many studies have confirmed the impact of pulmonary rehabilitation on the increase in functional fitness of patients with chronic respiratory diseases and on the improvement in their health-related quality of life [3,11,12]. Metanalyses show that pulmonary rehabilitation improves static and dynamic balance in patients with COPD, measured by clinical balance tests. Moreover, pulmonary rehabilitation combined with balance training, including strength training, endurance or walking exercises, aquatic exercises, exercises with neuromuscular electrical stimulation and specific balance training, in addition to traditional treatments, showed greater benefits than the conventional treatments based on improving respiratory symptomatology [4,13].

Postural equilibrium involves the coordination of movement strategies to stabilise the centre of body mass during disturbances in stability and is a complex motor skill based on the interaction of dynamic sensorimotor processes [14]. A systematic review by Moore and Backer [15] on studies of older adults found that the Four Square Step Test (FSST) may be an effective and valid tool for measuring dynamic balance and a participant’s fall risk [15]. One of the forms of treatment in respiratory system diseases using the natural underground environment is speleotherapy, otherwise known as subterraneotherapy. The studies carried out so far indicate the effectiveness of speleotherapy in the treatment of diseases of the upper respiratory tract, such as chronic rhinitis and paranasal sinusitis [16] and of the lower respiratory tract, such as COPD [17] and asthma [18] and are associated with an improvement in the functional fitness of older adults with respiratory dysfunction [19]. Subterranean, pulmonary rehabilitation combined with endurance and strength training, offered in our study to the elderly patients with chronic respiratory diseases, included regular walking on the uneven mining pavements, which activates balance via vestibular and proprioceptive system stimulation as well as walking training. The main therapeutic factors in speleotherapy are the air quality and stability of the microclimate. In the ‘Wieliczka’ Salt Mine Health Resort, the microclimate is characterized by low temperature (12.9–14.5 °C) and moderate/high relative humidity (60–75%), high concentration of minerals, ionization, very low level of dust, pollution and bioaerosol. The process of air purification occurs while the air travels through the ventilation system formed by salt tunnels and chambers with NaCl walls and results in an exceptionally low concentration of respiratory particulate matter (7 μg/m^3^) in the underground air [20,21,22]. Due to the constantly growing demand for effective methods of rehabilitation for the elderly, there is a need to conduct research on different physical exercise proposals, which influence the functional performance of older adults. Therefore, the question is whether dynamic balance performance and chest mobility of older adults can be expected to improve after a pulmonary rehabilitation combined with endurance and strength training intervention in the subterranean atmosphere of the salt mine.

## 2. Aim

The aim of the study was to assess the dynamic balance and chest mobility of older adults participating in speleotherapy combined with pulmonary rehabilitation, endurance and strength training.

## 3. Material and Methods

Criteria for inclusion in the research project:

1. Age—65 years and above
2. Obtaining a min. 10 points in the SPPB (Short Physical Performance Battery) test assessing the risk of disability in the elderly [23].
3. Indication for pulmonary rehabilitation with the use of subterraneotherapy methods (chronic respiratory disease) confirmed during qualification by a doctor.
4. Having no contraindication for speleotherapy (i.e., cancer, immunosuppressive treatment, severe circulatory failure, clinically active infectious diseases, rheumatic and degenerative diseases of the osteoarticular system, ischemic heart disease with unstable angina or arrhythmia, depression and claustrophobia syndromes, uncontrolled hypertension and unstable diabetes [24]) confirmed during qualification by a doctor.
5. Written, informed consent to participate in the project.

An assessment of functional fitness is required due to the specific location of the underground health resort: 135 m below the ground, 700 m distant from the descent shaft. Conditions taken as an indication for pulmonary rehabilitation combined with endurance and strength training and speleotherapy in the study group included 2 groups of participants with chronic diseases of the lower respiratory tract (59%, 26 patients) such as: bronchial asthma, COPD and bronchiectasis, as well as chronic diseases of the upper respiratory tract (41%, 18 patients) such as: sinusitis, pharyngitis and laryngitis [25]. This study was approved by the Regional Medical Ethics Board of Physicians in Krakow, Poland (pol. Okręgowa Izba Lekarska w Krakowie) No. 40/KBL/OIL/2018.

**Figure 1:**
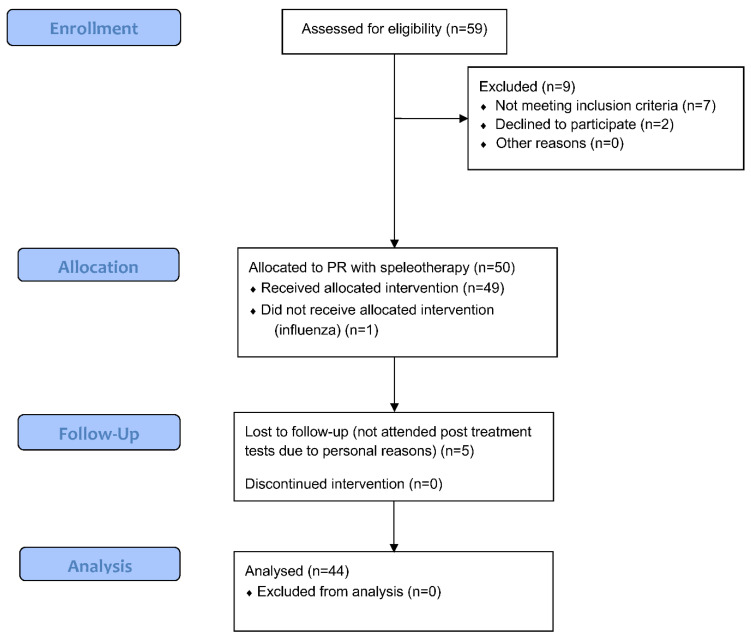
A flow diagram of the patients (www.consort-statement.org, accessed on 14 September 2022).

The study group consisted of 50 older adults (34 women and 16 men) with chronic respiratory diseases who participated in a 3-week pulmonary rehabilitation programme combined with endurance and strength training, in the underground salt chambers in the ‘Wieliczka’ Salt Mine Health Resort. [A study flow is presented on Figure 1].

During the study, 5 women withdrew from the project after the rehabilitation and treatment camp (no possibility to apply for final examinations) and 1 discontinued treatment in the underground salt chambers due to influenza. The test group for the eventual trial included 44 older adults aged 65 to 77 years (mean 68.8 ± 2.9 years). Their height ranged from 150 to 179 cm (mean 163.3 ± 8.0 cm), body weight from 65 kg to 103 kg (mean 76.0 ± 11.4 kg) and Body Mass Index (BMI) from 20.4 to 37.4 kg/m^2^ (mean BMI 28.5 ± 3.6). The mean age of 28 women was 68.5 ± 3.2 years and of 16 men 69.4 ± 2.5 years. The mean BMI of women was 28.4 ± 3.8 and for men 28.6 ± 3.5. Among comorbidities, we registered back pain in sixteen women and four men (45% of patients), lower limb pain in seven women and two men (20% of patients) and upper limb pain in six women and three men (20% of patients). Fourteen women registered between one and ten falls and six men between one and three falls in the last five years.

### 3.1. Study Design

After qualification by the medical doctor all persons included in the older adults study underwent the FSST and the chest mobility tests with a measuring tape by a trained research team immediately before and after their stay in the rehabilitation and treatment programme.

### 3.2. Four Square Step Test

**Figure 2:**
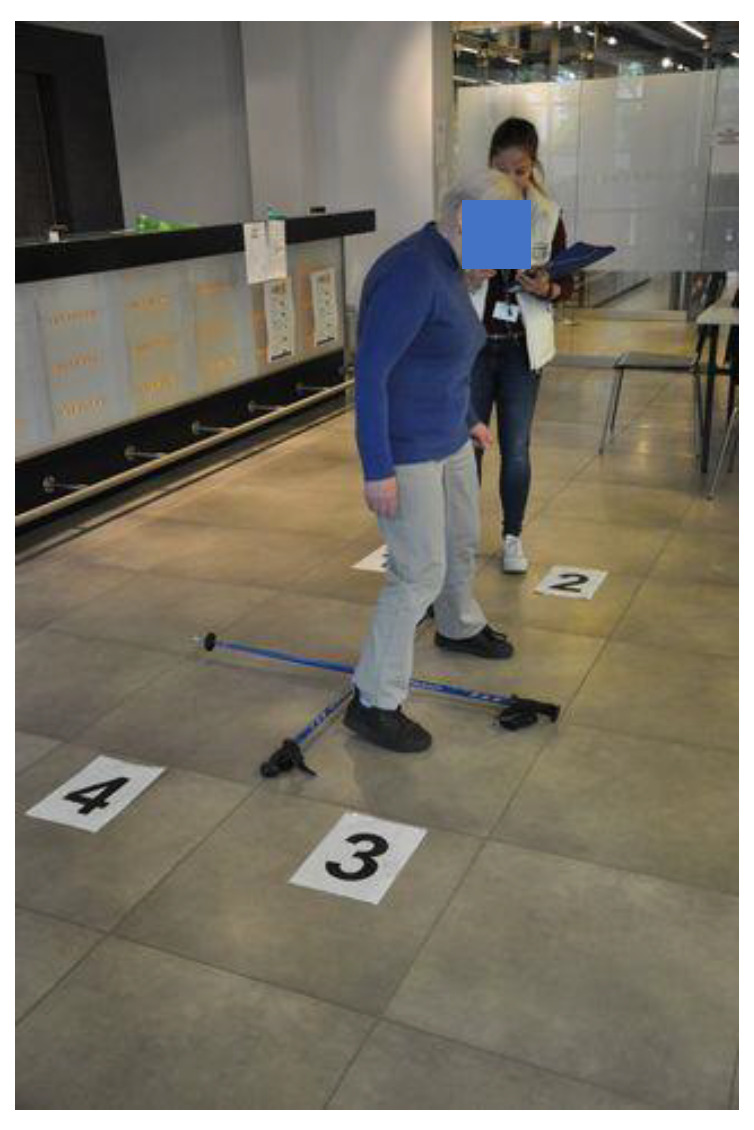
FSST performance with time measurement.

The Four Square Step Test (FSST) was used to assess the balance and body coordination of the subjects, according to the recommended procedure, which consisted of moving in different directions (front, back, right, left) while overcoming a small obstacle (4 sticks, 90 cm long, joined together in the shape of a cross, delimiting the fields marked with numbers from 1 to 4). Initially, the sequence of the task performed by the examiner was shown (squares 1-2-3-4-1-4-3-2-1), then the test was performed by the subject without time measurement and 2 tests were performed with the time of their completion (Figure 2). The examined person was verbally motivated to perform the test in the shortest possible time and the sample whose execution time was shorter was taken for analysis. The time needed to perform the FSST test was approximately 5 min. As reported by Mutchie et al. [26] FSST was developed by Dite and Temple [27] as a quick and easy-to-administer test for community-dwelling older adults, testing balance and motor planning to predict fall risk. The FSST has superb sensitivity (92%), specificity (93%), positive (86%) and negative predictive value (96%) for predicting multiple versus non-multiple fallers. The reliability and validity of the FSST compared with other mobility and balance tests (i.e., 6-Minute Walking Test (6 MWT), Timed Up-and-Go (TUG) and Step Test) were assessed across multiple disease conditions including stroke, Parkinson’s disease, muscular sclerosis, traumatic brain injury and vestibular dysfunction. In the study by Yazici et al. [28] aiming to evaluate balance in patients with chronic obstructive pulmonary disease with practical tests it was concluded that FSST and TUG had the highest correlation with the Berg Balance Scale (BBS), a time consuming but widely used test that can be applied for the assessment of bodily balance status among patients with COPD in outpatient clinics. Moore and Barker [15] in their systematic review about the validity and reliability of the FSST in different adult populations showed the concurrent validity of this test in nine of the studies with moderate to strong correlations. Excellent Intraclass Correlation Coefficients were found between physiotherapists carrying out the tests (ICC = 0.99) with good to excellent test–retest reliability shown in nine of the studies (ICC = 0.73–0.98).

### 3.3. Chest Mobility Test

**Figure 3:**
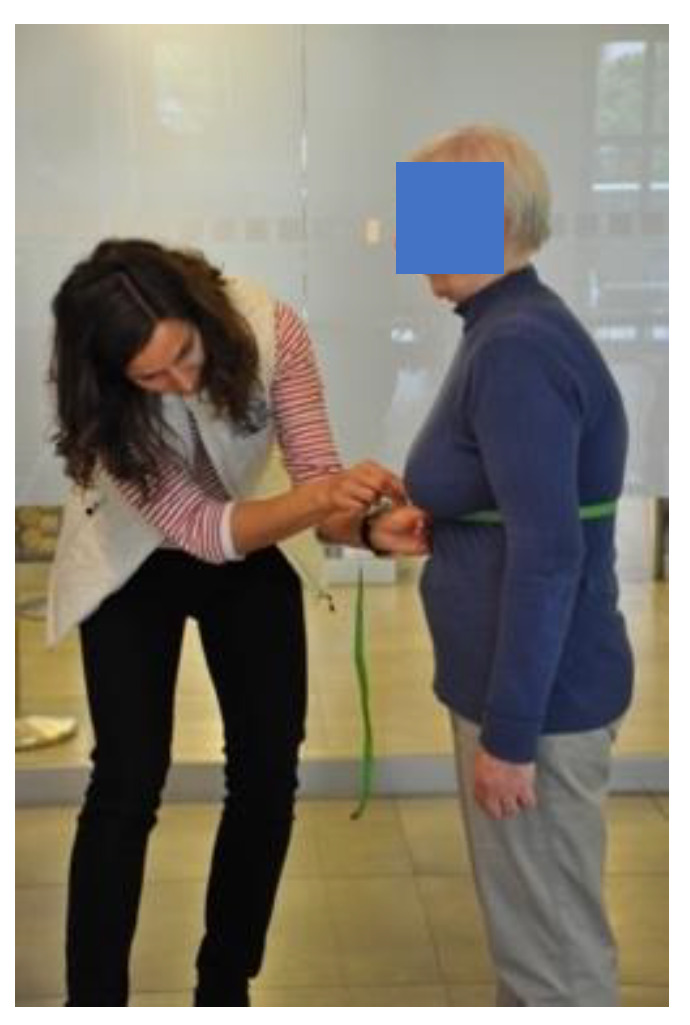
Circumferential chest expansion measurement.

During the examination, the patients stood dressed in thin, close-fitting clothing with their upper limbs loosely positioned along the body. The measurement was made 3 times at the height of the xyphoid process of the sternum and the height of the 10th spinous process (Figure 3). The person was motivated to perform maximum inhalation and exhalation [29,30,31]. The difference in circuit measurement at maximum inhale and exhale was recorded. The results of the older adult chest mobility before and after the rehabilitation and treatment stay were compared to the standards set for the circumferential chest expansion, regarding the age and sex of the patients [32]. As reported by Sharma et al. chest wall mobility measurement showed very good intraclass correlation coefficients (intratester reliability as 0.85 to 0.97 and intertester reliability as 0.93 to 0.97) in a study with 22 patients with ankylosing spondylitis and 25 healthy subjects. It was concluded that as reliability was good chest expansion measurement can be used for monitoring disease progression and efficacy of intervention with confidence within tester and between testers [33,34].

### 3.4. Speleotherapy

Speleotherapy refers to a special type of climatotherapy based on the use of natural factors in the underground environment to treat chronic respiratory diseases [35]. Speloetherapy is otherwise known as subterraneotherapy or Skulimowski method since Professor Mieczyslaw Skulimowski became the first official physician of the ‘Wieliczka’ Salt Mine and started regular treatment of patients in the salt chambers, initiating a new field of medicine utilizing the environmental benefits occurring underground. Indications for treatment with this method include upper and lower respiratory tract diseases, with particular emphasis on chronic obstructive pulmonary disease (COPD), recurrent rhinitis and inflammations of the sinuses, throat, larynx, chronic bronchitis, pneumonia, allergic diseases and bronchial asthma [36].

### 3.5. Pulmonary Rehabilitation Combined with Endurance and Strength Training in Underground Salt Chambers

The study was conducted between March 2018 and December 2019. An outpatient pulmonary rehabilitation (PR) was conducted 135 m below the Earth’s surface, for a period of 3 weeks (6 h a day for 5 days a week) in the ‘Wieliczka’ Salt Mine Health Resort (Table 1). PR programme combined with subterraneotherapy in the ‘Wieliczka’ Salt Mine is performed 5 days/week, while outpatient PR programmes commonly meet only 2 or 3 days/week [3]. Since the standard duration of the PR programme in the ‘Wieliczka’ Salt Mine is 3 weeks (and can be prolonged individually according to medical recommendations) the authors decided not to change the organization (duration and intensity) of the PR in the study protocol. Some research [17] shows the effectiveness of 3-week subterranean pulmonary rehabilitation in COPD patients. The main aims of the pulmonary programme in subterranean conditions included: increase in physical capacity, improvement in the patients’ general condition and health-related quality of life, reduction in chronic respiratory symptoms and the promotion of long-term adherence to health-enhancing behaviours. The PR programme included endurance training, strength training, breathing exercises, neuro-orthopaedic activity-dependent plasticity (N.A.P.) therapy techniques, education and relaxation.

### 3.6. Statistical Analysis

The sample size calculation (margin of error 10% and 90% confidence level) was 67 persons but a sample of the possible size was collected. The statistical power calculation with the use of PQStat 1.8.4. programme (PGStat Software Poznań/Plewiska, Poland) based on the results of FSST was 0.971 and the chest mobility was 0.92. Comparison of the values of quantitative variables in repeated measurements (before and after the rehabilitation programme) was performed with the Student’s *t*-test for dependent samples since the normal distribution of variables was confirmed by Shapiro–Wilk test. The Chi-square test of independence was used to assess the significance of differences before and after the programme of rehabilitation and treatment. The analyses were carried out using Statistica 13. Quantitative data are presented as mean (SD), while qualitative data are presented as N (%). A 5% level of statistical significance was chosen to minimise potential Type 1 errors.

## 4. Results

The average time needed to perform FSST decreased significantly (*p* ≤ 0.05) from 10.2 ± 1.9 cm before the stay to 9.1 ± 1.7 cm after the stay. For patients with lower respiratory tract disorders, the average decrease was 0.8 s and with upper respiratory tract disorders, 1.5 s (*p* ≤ 0.05) (Table 2).

The average chest circumference difference between the maximum inhalation and exhalation assessed in a free-standing position increased significantly (*p* ≤ 0.05) from 4.5 ± 5.5 cm before the stay to 5.4 ± 2.8 cm after the stay. Both for patients with lower and upper respiratory tract disorders, the average increase in chest mobility was 0.9 cm (*p* ≤ 0.05) (Table 3).

Before the rehabilitation in the underground salt chambers, half of the results (50%, 22 patients) were below the norm in the assessment of chest mobility between maximal inhale and exhale. After the stay, the share of people with normal chest mobility increased in the study group according to the standards established by Moll and Wright [32] from 50% to 66%, but the change was not statistically significant (*p* ≥ 0.05) (Table 4).

## 5. Discussion

In 2020, the World Health Organisation (WHO) published guidelines on physical activity and sedentary behaviour, which recommend at least 150 to 300 min of moderate to vigorous aerobic activity once a week for all adults, including people with chronic diseases or disabilities. Older adults (aged 65 years or older) were advised to add activities that emphasize balance and coordination, as well as muscle strengthening, to help prevent falls and improve health [37]. Moreover, the WHO recommends strategies for raising awareness on the risk of air pollution, as well as available solutions that can be implemented to mitigate the risks of exposure to air pollution [38]. Therefore, performing physical training, especially by older adults with respiratory dysfunction in a microbiological and palynological clean environment, seems to be highly justified. Speleotherapy includes breathing exercises in the pure environment of caves or mine chambers with their stable air temperature and moderate to high humidity in the presence of sodium, potassium, magnesium and calcium and in the absence of airborne pollutants and pollen [39]. Our study is one of the few in which the mobility of the chest was assessed in relation to the normative values and the dynamic balance of patients treated with the use of pulmonary rehabilitation in the conditions of underground salt workings. In the studies of LaPier et al. [40], with men and women (n = 120) without impairment, aged 20–70+ years, significant linear regressions between age and chest expansion were found [40]. In our study, following a pulmonary rehabilitation programme combined with endurance and strength training in underground conditions, we observed a significant increase in the mean difference in chest circumference between maximum inspiration and exhalation in older adults with both upper and lower respiratory tract disorders; however, this research did not include the control group with the same treatment overground. The result obtained differs from the results of the pilot project involving 22 elderly people. In this study, an increase in functional fitness measured with the Fullerton test was observed after a rehabilitation and treatment stay in the conditions of underground salt chambers in all fitness tests, except for the flexibility of the upper body [19]. The obtained outcomes resulted in the implementation of changes in the training programme, including the intensification of activities aimed at three-dimensional diaphragm movements and chest mobility. A study in adults with COPD [34] found that chest measurements taken with a measuring tape have good intra- and interrater reliability and reproducibility in healthy non-smokers, healthy smokers and COPD subjects and correlated with lung function parameters measured by a spirometer. Circumferential chest expansion may provide indirect information on lung function but interpretation with caution is required when considering implementation into a clinical setting. Chest wall excursion was used in the study of Ekstrum et al. [30] to assess a 6-week-long, performed-twice-a-day home exercise programme with thirty-seven volunteers (mean age 80.5 years). It was observed that after the rehabilitation, community-dwelling older adults participating in a 6-week stretching and respiratory exercise programme demonstrated improved chest mobility. In our observation before the rehabilitation programme, the difference in chest circumference between maximum inspiration and exhalation was, for half of the respondents, within the normative values given by Moll and Wright [32]. As a result of the intervention carried out in the conditions of underground salt chambers, chest mobility increased significantly, for patients with both lower and upper respiratory tract disorders. It would be advisable to establish current standards regarding normative values for chest mobility, with a division into gender and age of the respondents.

Fear of falling due to the feeling of lack of dynamic stability of the body during everyday activities, such as walking, dressing and reaching for objects, affects breathing changes because, in a situation of danger, we breathe faster and shallower. According to the study of Mutchie et al. [26], completing the FSST in more than 15 s has been found to be indicative of increased fall risk according to normative data. In our study, all elderly participants achieved results lower than 15 s in the FSST. Ventilation and posture are interrelated [3] and diaphragmatic breathing re-education is indicated in equilibrium training. The basis for restoring functional and motor fitness is the optimization of breathing. Maintaining a stable and efficient anti-gravity position that man has developed in the course of evolution requires constant balancing of the body’s centre of gravity during continuous dynamic breathing processes based on the mobility of the chest, diaphragm and internal organs of the body. Therefore, in comprehensive pulmonary rehabilitation combined with endurance and strength training, conducted in the conditions of underground salt chambers, the control of breathing and posture is taken into account, including activities to optimize the settings of the body’s diaphragm system (tentorium cerebelli, tongue, thoracic outlet, thoracic diaphragm and pelvic floor) through their myofascial connections [40].

It would be interesting to conduct research on postural alignment in people with respiratory problems based on the inter-relationship of alignment and position of the thoracic and pelvic blocks proposed by Collins et al. [41], using the Saliba Postural Classification System. Guidelines for pulmonary rehabilitation from the American Thoracic Society/European Respiratory Society recommend balance testing [3]. In our study, a significant increase in motor coordination and dynamic balance, as measured by the FSST test, was noted after a pulmonary rehabilitation programme, including breathing exercises and diaphragm auto-training, taking into account its three-dimensional mobility with respiratory movements. It is known that the diaphragm affects the breathing and posture control through anatomic, fascial and neurologic networks [42]. The study by Kocjan et al. [43], with the participation of patients with a diagnosis of lung cancer and patients undergoing lung resection, shows that the diaphragm plays an essential role in static balance maintenance and, like other previously described functions, this balance function is also inextricably linked to breathing. Impairment of diaphragm function manifested by a decrease in muscle thickness and movement restriction is strongly associated with balance disorders in a clinical sample and among healthy subjects. According to Horak [14], postural orientation involves the active control of body alignment and tone with respect to gravity, support surface, visual environment and internal references and is no longer considered simply as a summation of static reflexes but, rather, a complex skill based on the interaction of dynamic sensorimotor processes.

In the studies by Cano et al. [44] it was shown that vertical perturbations generate unique balance-correcting muscle activations [44]. The improvement in the dynamic balance after treatment in the conditions of the underground salt chambers could have been influenced by the inclusion of walking training on uneven ground (mine pavement) over a distance of about 700 m in the pulmonary rehabilitation programme combined with endurance and strength training. Participants in our own study marched twice a day on this uneven surface, in conditions of increased stimulation of the proprioception system due to underground, artificial lighting and limitations for collecting information from the visual system. In research conducted by Smith et al. [45], 11 participants diagnosed with COPD and asthma attended a 8-week out-patient pulmonary rehabilitation programme, which involved customized exercise prescription directed by a physiotherapist. Subjects were also given a walking programme to improve cardiovascular fitness. The distance and time to walk was determined from the results of a 6 min walk test performed by the subjects during the initial assessment. To assess the balance of subjects, the FSST was used because, apart from its high inter-rater and test–retest reliability [27], it also involves a greater challenge to balance in the medial-lateral direction, which is considered critical for fall risk. After participation in the pulmonary rehabilitation programme combined with endurance and strength training, subjects’ performance improved on the FSST from 9.3 (7.2–14.2) to 8.7 (7.4–10.2) seconds (*p* = 0.050). The average decrease was 0.6 s and is similar to the result obtained in our study (0.8 s). Older adults with chronic respiratory diseases can improve their dynamic balance and chest mobility with pulmonary rehabilitation combined with endurance and strength training in the underground salt chambers. Future randomized controlled trials are needed to evaluate the effect of speleotherapy in pulmonary patients in older age.

## 6. Limitations

The limitations of the studies are as follows:

(1) Lack of randomization.
(2) Lack of control group that would implement the same programme overground.
(3) Lack of a representative sample size based on the calculation and research blinding in the initial and final evaluation protocols.
(4) Lack of other performance or health-related measurements, such as body mass, following the 3-week training programme.

Nevertheless, the obtained results confirm that speleotherapy with pulmonary rehabilitation, endurance and strength training can contribute to improvements in dynamic balance and increasing the mobility of the chest in older adults.

## 7. Conclusions

Speleotherapy combined with pulmonary rehabilitation, endurance and strength training increased the dynamic balance and chest mobility of older adults with chronic respiratory diseases, as measured by the FSST and circumferential chest expansion assessment.

## Figures and Tables

**Table 1:** Day schedule of the rehabilitation and treatment stay at the “Wieliczka” Salt Mine Health Resort in Wieliczka.

Time	Timetable
15–20 min	Descent to the mine using a shaft, 700 m group walk to the treatment chambers on uneven surface of miners’ route
30–60 min	Break
30 min	Endurance training: aerobic exercise with or without tools such as sensory balls, elastic bands etc.
60–90 min	Break
30 min	Breathing exercises including breath control strategies, respiratory muscles training, resistive training, chest elasticity exercises with or without tools such feathers, pipes etc.
60–90 min	Break
35 min	Strength training with or without tools/Specialized training using N.A.P. therapy, relaxation methods and others.
30–60 min	Break or educational panel run by medical staff
15–20 min	700 m group walk to the shaft and ascent to the surface on uneven surface of miners’ route

**Table 2:** FSST execution time before and after a pulmonary rehabilitation programme combined with endurance and strength training in the underground environment.

	Before Treatment [s]		After Treatment [s]		Difference [s]	t	*p*
	Mean	SD	Mean	SD			
Total	10.2	1.9	9.1	1.7	1.1	3.9445	0.0003
Lower respiratory tract diseases	10.3	1.8	9.5	2.1	0.8	2.1495	0.0415
Upper respiratory tract diseases	10.0	2.0	8.5	1.0	1.5	3.7516	0.0016

**Table 3:** Chest mobility before and after pulmonary rehabilitation programme combined with endurance and strength training in the underground environment.

	Before Treatment [cm]		After Treatment [cm]		Difference [cm]	t	*p*
	Mean	SD	Mean	SD			
Total	4.5	2.5	5.4	2.8	−0.9	−3.3441	0.0017
Lower respiratory tract diseases	4.6	2.7	5.5	3.0	−0.9	−2.4808	0.0202
Upper respiratory tract diseases	4.3	2.3	5.2	2.4	−0.9	−2.1902	0.0427

**Table 4:** The results of the study group’s chest mobility within the norms by Moll and Wright [32] before and after the treatment stay in the ‘Wieliczka’ Salt Mine Health Resort.

	Reference to Norms	Before Treatment		After Treatment		χ^2^	df	*p*
		N	%	N	%			
Total N = 44	Below the norms	22	50	15	34	2.2851	1	*p* > 0.05
	Within the norms	22	50	29	66			
Lower respiratory tract disorders N = 26	Below the norms	12	46	9	35	0.7189	1	*p* > 0.05
	Within the norms	14	54	17	65			
Upper respiratory tract disorders N = 18	Below the norms	10	56	6	33	1.8000	1	*p* > 0.05
	Within the norms	8	44	12	67			
